# Quasimetagenomic source tracking of *Listeria monocytogenes* from naturally contaminated ice cream

**DOI:** 10.1186/s12879-019-4747-z

**Published:** 2020-01-29

**Authors:** Andrea Ottesen, Padmini Ramachandran, Yi Chen, Eric Brown, Elizabeth Reed, Errol Strain

**Affiliations:** 1 Center for Food Safety and Applied Nutrition (CFSAN), FDA 5001 Campus Drive, College Park, MD 20740 USA; 2 Center for Veterinary Medicine (CVM), FDA 8301 Muirkirk Rd, Laurel, MD 20708 USA

**Keywords:** Quasimetagenomic, (qMGS), Quasimetagenome, Metagenomic, Metagenome, Source tracking, *Listeria monocytogenes*, Ice cream, Microbiological enrichment

## Abstract

**Background:**

The more quickly bacterial pathogens responsible for foodborne illness outbreaks can be linked to a vehicle of transmission or a source, the more illnesses can be prevented. Whole genome sequencing (WGS) based approaches to source tracking have greatly increased the speed and resolution with which public health response can pinpoint the vehicle and source of outbreaks. Traditionally, WGS approaches have focused on the culture of an individual isolate before proceeding to DNA extraction and sequencing. For *Listeria monocytogenes (Lm),* generation of an individual isolate for sequencing typically takes about 6 days. Here we demonstrate that a hybrid, “quasimetagenomic” approach ie; direct sequencing of microbiological enrichments (first step in pathogen detection and recovery) can provide high resolution source tracking sequence data, 5 days earlier than response that focuses on culture and sequencing of an individual isolate. This expedited approach could save lives, prevent illnesses and potentially minimize unnecessary destruction of food.

**Methods:**

Naturally contaminated ice cream (from a 2015 outbreak) was enriched to recover *Listeria monocytogenes* following protocols outlined in the Bacteriological Analytic Manual (BAM). DNA from enriching microbiota was extracted and sequenced at incremental time-points during the first 48 h of pre-enrichment using the Illumina MiSeq platform (2 by 250), to evaluate genomic coverage of target pathogen, *Listeria monocytogenes*.

**Results:**

Quasimetagenomic sequence data acquired from hour 20 were sufficient to discern whether or not *Lm* strain/s were part of the ongoing outbreak or not. Genomic data from hours 24, 28, 32, 36, 40, 44, and 48 of pre-enrichments all provided identical phylogenetic source tracking utility to the WGS of individual isolates (which require an additional 5 days to culture).

**Conclusions:**

The speed of this approach (more than twice as fast as current methods) has the potential to reduce the number of illnesses associated with any given outbreak by as many as 75% percent of total cases and potentially with continued optimization of the entire chain of response, contribute to minimized food waste.

## Background

Next generation sequencing (NGS) technologies have revolutionized our ability to source track pathogens such as *Salmonella enterica* [1]*, Listeria monocytogenes* [2], *Escherichia coli* [3]*,* and many others by facilitating the whole genome sequencing (WGS) of these small bacterial genomes in as little as 20h hours. Sequence data is organized using pipelines that generate matrices of single nucleotide polymorphisms (SNPs) that can be used with a variety of algorithms to infer phylogenetic relationships. This approach can differentiate highly clonal strains within serovars that differ by as few as 1 to 29 nucleotides [4]. GenomeTrakr is the first of its kind network, organized by the U.S. Food and Drug Agency (FDA), the National Center for Biotechnology Information (NCBI) and the Centers for Disease Control (CDC), to standardize and coordinate the collection and sharing of WGS data with the goal of rapidly identifying origins of pathogens associated with illness outbreaks [5]. The biggest time constraint for WGS source tracking is simply the culturing of the bacteria from which DNA is to be extracted and sequenced. Typical recovery of *Lm* from a food source, as described in the U.S. Food and Drug Administration (FDA) *Bacteriological Analytical Manual* (BAM) begins with 48 h of pre-enrichment, followed by incubation on selective agars (another 48 h), growth on nutrient agars (24 h) and then confirmation (3 to 24 h) of individually selected colonies [6]. Time commitment for the full protocol is between120 h and 144 h (~ 6 days). Culture independent approaches are an alternative but still require financially and computationally impractical amounts of sequence data to achieve sufficient genomic coverage of low abundant targets that occur amidst a complex microbial ecology.

To evaluate hybrid options for improving the speed of source tracking, we sequenced DNA from enrichment microbiomes (every 4 hours) using short read (Illumina Miseq) sequencing. Using naturally contaminated ice cream previously linked to an outbreak of listeriosis that resulted in three deaths [4], shotgun data from incremental time-points over the first 48 h of pre-enrichment were evaluated to identify when *Lm* genomic data with source tracking utility could be obtained from the quasimetagenomes. Because the word “metagenomics” refers to true culture independent (CI) genomics [7], we use the term “quasimetagenomics” [8] to describe sequence data from pre-enrichments, which by their nature, are only partial selections of the environmental pan-genome.

## Methods

### Enrichment, DNA extraction and sequencing

#### Enrichment

Homogenized ice cream scoop samples were added to Buffered Listeria Enrichment Broth (BLEB) according to the specifications for *Lm* recovery outlined in the BAM [6]. Four replicates of negative (no ice-cream) and positive controls (*Lm* cells) were assayed at all time-points.

#### DNA extraction

DNA was extracted using DNeasy Blood and Tissue kit (Qiagen) following the protocol for Gram-positive bacteria with minor modifications: 1.5 ml of the culture was pelleted (5000×g, 15 min) and the pellet resuspended in 200 μL of enzymatic lysis buffer containing 20 mM Tris-HCl (pH -8.0), 2 mM Sodium EDTA, 1.2% Triton X- 100, 20 mg/ml of lysozyme. The samples were incubated for 60 min at 37 °C.

#### Library preparation

Libraries were prepared with Nextera XT (Illumina) according to the manufacturers specifications. For the first and second enrichments, approximately 16 libraries (independent replicates) were multiplexed per run (total of 3 runs) using Illumina MiSeq V2 (2 by 250).

#### Bioinformatic analyses

WGS analyses were performed using the CFSAN SNP pipeline 0.6.1 [9, 10]. Raw reads from each replicate from all hours were mapped to the complete genome of CFSAN029793, (BioSample SAMN03386937) using default settings within Bowtie2 v2.2.2 [11]. A resulting BAM (xyz) file was sorted using Samtools v1.3.1 [12] and a pileup file for each isolate was produced. These files were then processed using VarScan2 v2.3.9 [13] to identify high quality variant sites, using the mpileup2snp option. Additional information about these procedures, e.g. codes and instructions, is available at https://github.com/CFSAN-Biostatistics/snp-pipeline. Phylogenetic trees were created using the Genetic Algorithm for Rapid Likelihood Inference (GARLI) [14]. Taxonomic profiles of enrichment microbiomes were assigned using CosmosID’s bacterial database and inhouse – k-mer based bacterial databases and pipelines.

#### Data availability

All data is available at NCBI associated with BioProject PRJNA370011.

## Results

### Evaluating genomic coverage of *Listeria monocytogenes* for trace-back utility

**Fig. 1:**
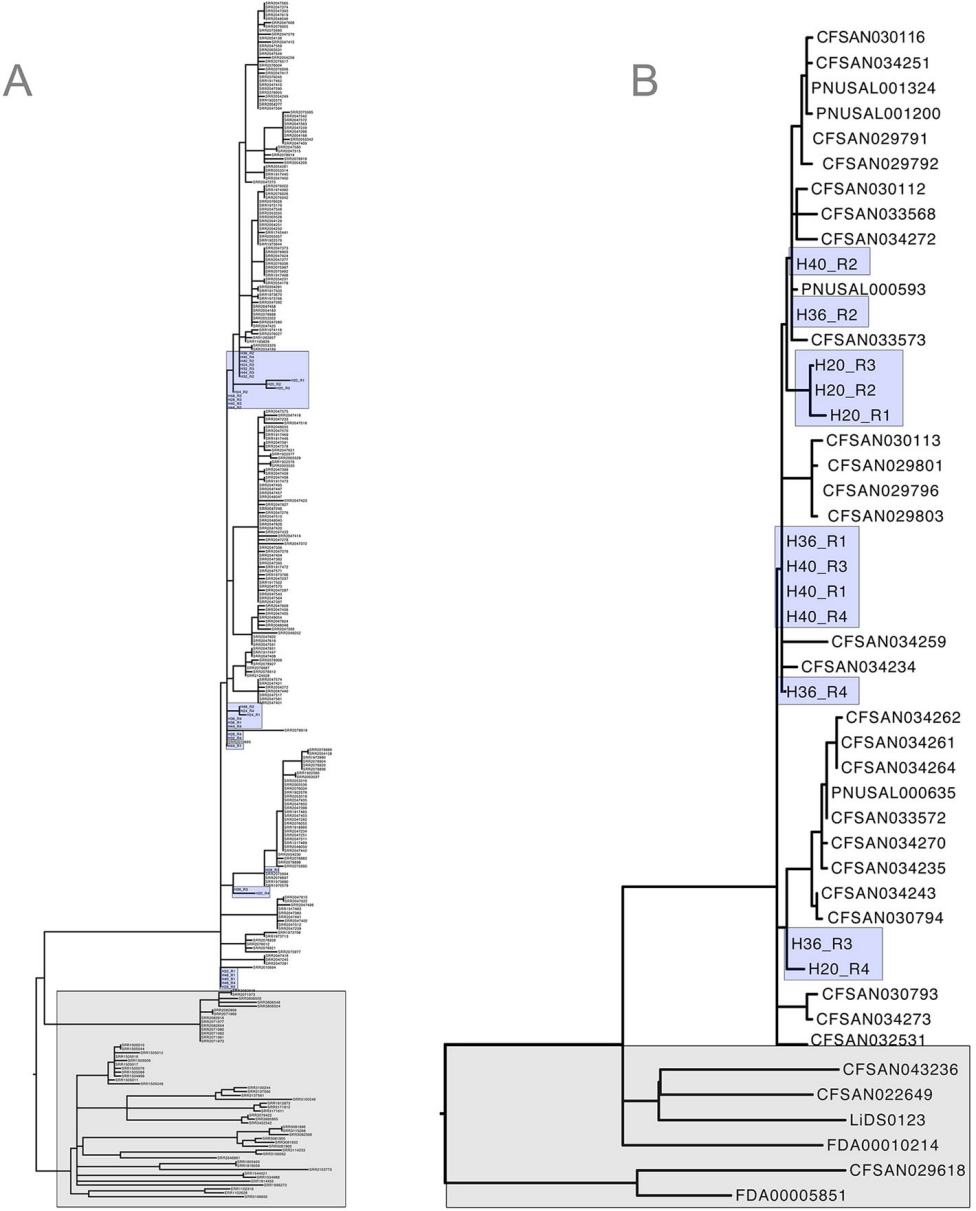
Full (**a**) and abbreviated (**b**) phylogeny of the NCBI outbreak cluster of *Listeria monocytogenes* (environmental and clinical) from contaminated ice cream. **a**. Phylogeny of the full representative set of the NCBI outbreak associated cluster (environmental and clinical isolates associated with the 2015 listeriosis outbreak linked to ice cream). *Listeria monocytogenes* genomic data from pre-enrichment microbiomes from hours: 20 to 40 are shown clustering with WGS of individual isolates (blue shading) (that took five days longer to obtain). The outgroup (grey shading) is comprised of closely related genomes of *Lm* not associated with the outbreak. **b**. A phylogeny of an abbreviated set of clinical and environmental isolates associated with the 2015 listeriosis outbreak linked to ice cream. WGS genomes from the full *Listeria monocytogenes* enrichment protocol are shown clustering with genomic data of *Listeria monocytogenes* from pre-enrichment microbiomes from hours 20, 36 and 40 (blue shading). The outgroup (grey shading) is comprised of closely related genomes of *Lm* not associated with the outbreak. A full list of all isolates, sources and accession numbers used for the full tree and the abbreviated tree is available in Additional file 1

At h 20, from an average of 2.4 million sequences per replicate, an average of 1.68x coverage was obtained for *Lm* genome/s from ice-cream enrichments (Table 1). This coverage was sufficient to identify whether or not the candidate strain/s of *Lm* were part of the outbreak cluster or not. Determination of inclusion or exclusion after only 20 h (plus sequencing time) in cases where a reference genome is available facilitates a greatly expedited ability to remove contaminated commodities from the food supply and protect safe products from costly holds. High quality draft *Lm* genomes were achieved with data from h 24 through h 48 with coverage (12.74 to > 52.75x) -shown for hours 20–40 in Table 1. *Lm* genomes from enrichments of all time-points from h 20 through h 48 were used with WGS genomes of environmental and clinical isolates from the full outbreak cluster (available at NCBI) (Fig. 1a). An abbreviated representational set of clinical, food and environmental isolates is also shown in Fig. 1b with quasimetagenomes from enrichments of hours 20, 36 and 40. A list of genomes comprising the complete outbreak is available in Additional file 1.

**Table 1:** Coverage statistics for *Listeria monocytogenes* from h 20 to h 40 of enrichments

Hour	Rep	Number of Reads	Reads Mapped to *L. mono*	Average Coverage
20	1	2.2E+ 06	1.66%	1.57X
20	2	2.6E+ 06	1.44%	1.62X
20	3	2.3E+ 06	1.27%	1.28X
20	4	2.7E+ 06	1.98%	2.23X
	Avg	2.4E+ 06	1.60%	1.68X
24	1	1.7E+ 06	11.82%	9.08X
24	2	1.7E+ 06	15.12%	12.03X
24	3	2.1E+ 06	18.07%	15.50X
24	4	2.3E+ 06	15.32%	14.34X
	Avg	2.0E+ 06	15.10%	12.74X
28	1	1.9E+ 06	51.80%	41.27X
28	2	1.5E+ 06	54.90%	33.99X
28	3	1.1E+ 06	51.74%	25.40X
28	4	1.0E+ 06	56.93%	26.83X
	Avg	1.4E+ 06	53.84%	31.87X
32	1	1.4E+ 06	87.44%	49.47X
32	2	9.1E+ 05	88.99%	34.97X
32	3	1.6E+ 06	78.56%	52.97X
32	4	1.4E+ 06	88.78%	54.78X
	Avg	1.3E+ 06	85.90%	48.05X
36	1	8.8E+ 05	96.14%	58.54X
36	2	6.2E+ 05	84.28%	37.91X
36	3	1.1E+ 05	89.32%	4.45X
36	4	1.1E+ 06	96.01%	63.27X
	Avg	6.7E+ 05	91.40%	41.04X
40	1	9.0E+ 05	97.78%	51.25X
40	2	4.1E+ 05	89.68%	26.50X
40	3	1.0E+ 06	76.92%	51.79X
40	4	1.4E+ 06	96.94%	81.45X
	Avg	9.4E+ 05	90.30%	52.75X

Hour, replication number, number of total reads, percentage of reads mapped to *Lm,* and *Lm* genome coverage statistics are shown for each of four independent replicates of ice cream enrichments from h 20 to h 40 of recovery enrichments

### Relative abundance of *Listeria monocytogenes* and other co-occurring genera

**Fig. 2:**
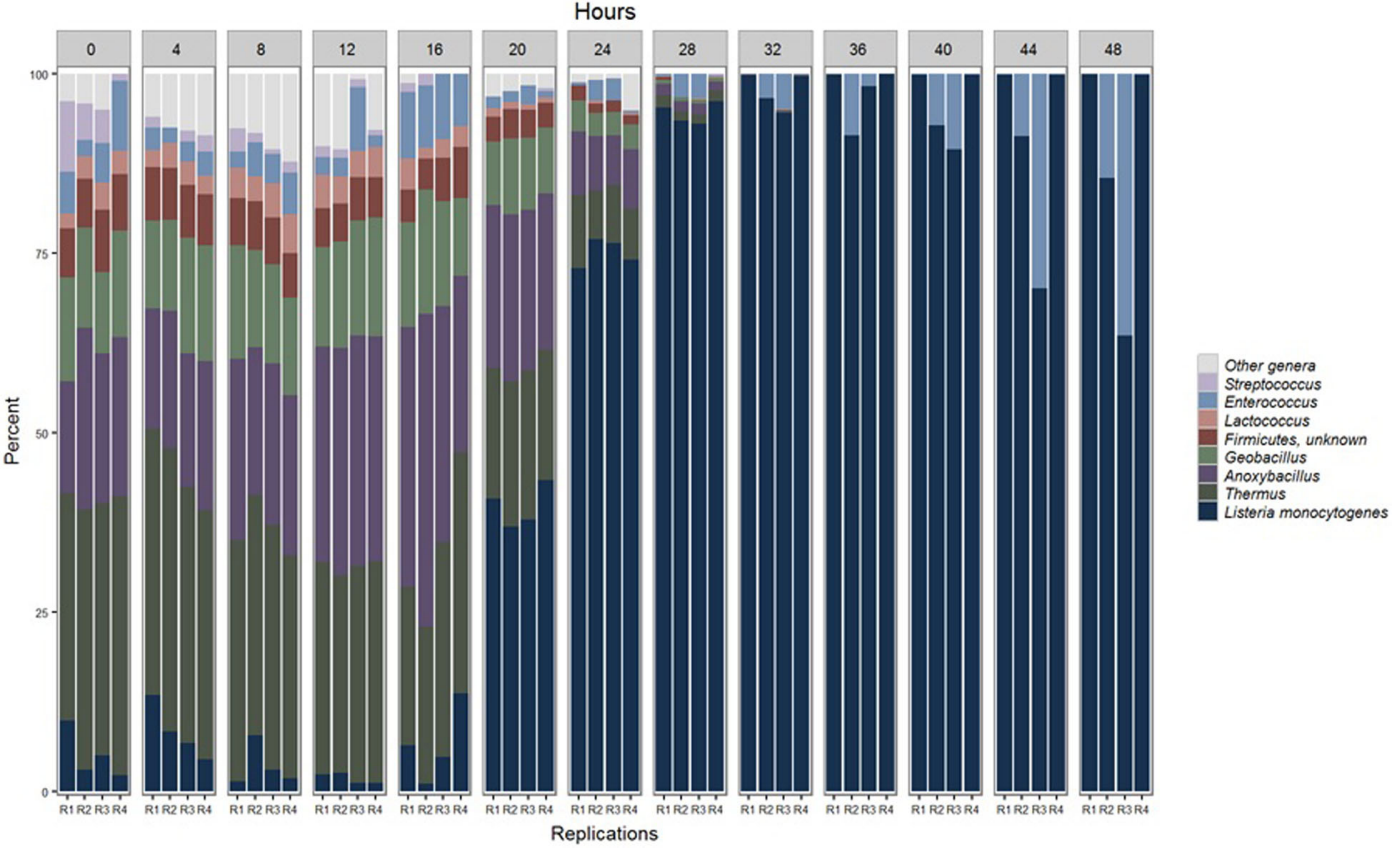
Relative abundance of *Listeria monocytogenes* and other co-enriching bacterial genera throughout the 48 h of pre-enrichment for recovery of *Lm* from ice-cream. From h 0 to h 48, at 4 h increments, the relative abundance of *Listeria monocytogenes* and other co-enriching bacterial genera that grow during recovery of *Listeria monocytogenes* from a dairy homogenate are described (*n* = 4) per time-point. Taxonomy was assigned using CosmosID with their bacterial database (Rockville, MD)

As microbiota from the naturally contaminated ice cream grew in the FDA BAM delineated media (BLEB), the following predominant bacterial genera were observed; *Thermus* (Thermales, Deinococcus-Thermus), *Anoxybacillus,* and *Geobacillus* (Bacillales, Firmicutes), and *Lactococcus, Enterococcus* and *Streptococcus* (Lactobacillales, Firmicutes) (Fig. 2). *Lm* remained at very low levels until h 20, introducing the possibility that the co-culturing Bacilli genera (*Anoxybacillus* and *Geobacillus*) play a role in inhibiting *Listeria monocytogenes* until it proliferates in h 20. This same phenomenon was also noted in previous work that focused on the description of the total ecology of enriching microbiota from naturally contaminated ice-cream using the three most commonly used enrichment protocols by; the International Organization for Standardization (ISO), the United States Department of Agriculture (USDA) and the FDA [15].

## Discussion

Metagenomic approaches have been accidentally and intentionally employed for decades to describe “black boxes” of human and environmental microbiota. These inquiries coupled with rapidly advancing NGS technologies have contributed to a renaissance in our understanding of numerous complex, previously poorly understood microbial ecologies. Focusing this approach on the “black box” of culture based pathogen recovery methods, many of which have not changed in 30+ years, provides novel insight to further expedite and optimize state of the art pathogen recovery methods. This applies both to the microbiological dynamics, ie; can we identify chemical, temperature or antibiotic inputs that would optimize recovery of targets by inhibiting competitors. Or, as presented here, by direct capture of genomic data of enriching targets from within the enrichment microbial community (quasimetagenome).

A true CI metagenomic approach was used to successfully identify strains of Shigatoxigenic *E. coli* O104: H4 linked to a 2011 outbreak that resulted in over 40 deaths [16]. This powerful demonstration relied on extremely high throughput sequencing and extensive bioinformatic analyses. The authors describe the exciting potential, while acknowledging the significant challenges that remain, such as “speeding up and simplifying workflows, reducing costs, and improving diagnostic sensitivity”. Thus, the application of metagenomics for source tracking is valuable for certain inquiries but is not yet practical as a rapid, inexpensive approach that can be implemented in field and public health laboratories. The high costs of generating and analyzing sufficient genomic data to distinguish between the highly clonal organisms responsible for many foodborne illnesses such as *E. coli*, *Salmonella enterica* and *Listeria monocytogenes,* when they occur as low abundant members of highly diverse ecologies, are still prohibitive for most operations. Thus, validation of the quasimetagenomic approach (culture and shotgun sequencing) presented here, offers a protocol that can be easily integrated into public health laboratories at no additional cost to current programs.

## Conclusions

The benefits achieved by the quasimetagenomic (qMGS) approach presented here, efficiently address previously described challenges of cost and speed, while maintaining diagnostic sensitivity. The source tracking phylogenetic success achieved by this experiment is particularly valuable because it validates the hybrid approach using *naturally contaminated* food samples. With modeled (*spiked in*) experiments, it is difficult to understand if results represent “real life” contamination dynamics or not. Quasimetagenomic sequencing can be applied to all culturable pathogens from all types of matrices (food or other). Ultimately this approach might have incredibly significant utility in hospital situations for rapid identification of strains causing septicemia. Temporal parameters, enrichment schema, and quantity of data necessary for optimal genomic coverage will be different for every pathogen, substrate and matrix. As shown here for source tracking *Listeria monocytogenes* from ice-cream the qMGS approach provides the exact same answer as WGS in a timeframe that when leveraged efficiently, could potentially eliminate the majority of illnesses associated with many outbreaks and potentially reduce unnecessary destruction of food.

## Supplementary information

**Additional file 1.** Annotation for phylogenetic trees of full and abbreviated NCBI 2015 listeriosis outbreak cluster linked to ice cream.

## Data Availability

All data is available at NCBI associated with BioProject PRJNA370011 https://www.ncbi.nlm.nih.gov/bioproject/PRJNA370011.

## Abbreviations

BAM: Bacteriolgical Analytical Manual
BLEB: Buffered *Listeria* enrichment broth
CDC: Centers for Disease Control
CFSAN: Center for Food Safety and Applied Nutrition
CI: Culture independent
DNA: Deoxyribonucleic acid
EDTA: Ethylenediaminetetraacetic acid
FDA: Food and Drug Administration
GARLI: Genetic Algorithm for Rapid Likelihood Inference
HCL: Hydrogen chloride, Hydrochloric acid
ISO: International Organization for Standardization
*Lm*: *Listeria monocytogenes*
NCBI: National Center for Biotechnology Information
NGS: Next generation sequencing
qMGS: Quasimetagenomic sequencing
SNP: Single nucleotide polymorphism
USDA: United States Department of Agriculture
WGS: Whole genome sequencing
